# YouTube Videos Provide Low-Quality Educational Content About the Anterolateral Ligament of the Knee

**DOI:** 10.1016/j.asmr.2024.101002

**Published:** 2024-09-12

**Authors:** Riccardo D’Ambrosi, Alessandro Carrozzo, Alessandro Annibaldi, Thais Dutra Vieira, Jae-Sung An, Benjamin Freychet, Bertrand Sonnery-Cottet

**Affiliations:** a IRCCS Ospedale Galeazzi–Sant’Ambrogio, Milan, Italy; b Dipartimento di Scienze Biomediche per la Salute, Università degli Studi di Milano, Milan, Italy; c Orthopaedic Unit, Sant’Andrea Hospital, University of Rome La Sapienza, Rome, Italy; d Centre Orthopédique Santy, FIFA Medical Center of Excellence, Hopital Mermoz, Groupe Ramsay, Lyon, France

## Abstract

**Purpose:**

To evaluate the reliability and quality of the educational content of YouTube videos about the anterolateral ligament (ALL).

**Methods:**

A standard search of the YouTube database was performed. All English-language videos were included for analysis. Video reliability was assessed with the use of the DISCERN tool, *Journal of the American Medical Association* (JAMA) benchmark criteria, and Global Quality Score (GQS). Data regarding the duration of the videos, date of publication, and number of likes and views were collected. Furthermore, videos were categorized based on video source (health professional, company, or private user), type of information (surgical technique, overview, or anatomy, radiology, and patient experience), and video content (education or patient experience/testimony).

**Results:**

A total of 94 videos were included in the analysis. Of these videos, 88 (93.6%) were published by health professionals, whereas 4 (4.3%) were published by companies and 2 (2.1%) were published by private users. Most of the videos were about surgical technique (57.4%), and almost all the videos (98.9%) had an educational aim, with the exception of 1 video that reported a patient experience (1.1%). The mean length of the videos was 648.4 ± 973.5 seconds, and the mean online period was 34.5 ± 27.0 months. The mean DISCERN score, JAMA score, and GQS were 32.9 ± 15.9, 1.5 ± 0.9, and 2.3 ± 1.0, respectively. Videos that provided an overview of the ALL were the best in terms of all quality scores and were significantly higher quality than videos about surgical technique and anatomy, radiology, and patient experience for all scores (*P* < .001). No difference was found between surgical technique and anatomy, radiology, and patient experience (DISCERN score, *P* > .99; JAMA score, *P* = .839; and GQS, *P* > .99).

**Conclusions:**

The educational content of YouTube videos about the ALL of the knee showed low quality and validity based on the DISCERN score, JAMA score, and GQS.

**Clinical Relevance:**

With the growing use of social media by patients to gather information about their medical conditions, it is crucial for orthopaedic health care providers to recognize the limitations of social media videos discussing the ALL as potential sources of knowledge for their patients.

Since the beginning of 2020, the entire world has been under conditions of temporary cessation of regular activities as a result of the coronavirus disease 2019 (COVID-19) pandemic. The worldwide outbreak has had a substantial impact on nearly all countries. To halt the transmission of the virus, extensive measures have been implemented to restrict social and professional activities.^1^

Specifically, when it comes to the field of education, the choice to completely shut down schools, partially shut down schools, or resume operations at schools should be based on a risk management approach. This approach aims to optimize the educational, well-being, and health advantages for students, teachers, staff, and the wider community while also working to prevent a recurrence of COVID-19 in the community.^2^

Since the year 2000, online education has emerged as a major area of study in the field of education, serving as a valuable addition to the traditional education model. As the online technology progresses, the software and hardware equipment needed for online teaching also evolves, hence influencing the effectiveness of online education. The networking process in medical education has become more advanced compared with other specialties. Online medical education has contributed to undergraduate and postgraduate training, as well as the ongoing professional development of doctors. After the emergence of the coronavirus, social factors have played a significant role in the growth of online education, leading to shifts in research focus and areas of interest.^3^, ^4^, ^5^

There has been increased patient interest in orthopaedic online searches in recent years because more than 50% of orthopaedic patients use social media or the internet for both work and personal communications.^6^ One of the most widely used websites for searching for medical information is YouTube (Alphabet, Mountain View, CA), which allows the sharing of video content and is accessed by more than 2 billion users.^7^ Previous studies have evaluated the reliability and quality of content on YouTube with respect to various orthopaedic pathologies, including shoulder instability, meniscal lesions, kyphosis, and femoroacetabular impingement, and treatments, including anterior cruciate ligament reconstruction (ACLR), revealing low quality and poor information.^8^, ^9^, ^10^, ^11^, ^12^ The purpose of this study was to evaluate the reliability and quality of the educational content of YouTube videos about the anterolateral ligament (ALL). The hypothesis of the study was that the educational quality of information regarding the ALL on YouTube would be low.

## Methods

This study focused on YouTube videos about the ALL of the knee and lateral extra-articular tenodesis (LET), and it was exempt from institutional review board approval. The following terms were used as keywords to search YouTube (May 10, 2024): “anterolateral ligament of the knee,” “ALL reconstruction,” “LET,” “lateral extra-articular tenodesis,” “Lemaire,” and “Segond fracture.” All the videos for each term were obtained. Irrelevant videos, non–English-language videos, duplicate videos, and videos with poor pronunciation were excluded from the study.

The duration of the videos, date of publication, and number of likes and views were retrieved. Furthermore, videos were categorized based on video source (health professional, company, or private user [defined as a patient or potential patient who was not involved in health care]), type of information (surgical technique, overview, or anatomy, radiology, and patient experience), and video content (education or patient experience/testimony). These classifications were made based on the data provided by the videos.

The quality and reliability of video content were assessed by 2 experienced knee surgeons using the DISCERN tool, *Journal of the American Medical Association* (JAMA) benchmark criteria, and Global Quality Score (GQS).^8^^,^^9^^,^^13^, ^14^, ^15^, ^16^, ^17^ The primary author (R.D.) analyzed each video regarding inclusion and exclusion criteria, separate and blinded from other authors who contributed to video scoring. The video links were presented to the observers in the form of a table, and the 2 observers (A.C. and A.A.) evaluated and scored the videos according to the DISCERN tool, JAMA criteria, and GQS.

Two independent physicians (J-S.A. and B.F.) experienced in knee arthroscopic surgery assessed each video for eligibility and content. Discrepancies regarding eligibility were addressed and discussed with the senior author (B.S-C.).

### Assessment Tools for Video Reliability, Validity, and Quality

##### Discern Instrument

The DISCERN tool is an assessment scale developed for patients and providers to assess the reliability and quality of information.^13^^,^^15^ The reliability and quality of information are characterized as follows: a score of 15 to 27 points denotes “very poor”; 28 to 38 points, “poor”; 39 to 50 points, “fair”; 51 to 62 points, “good”; and 63 to 75 points, “excellent.” The DISCERN tool is freely accessible at http://www.discern.org.uk.^18^

##### JAMA Benchmark Criteria

The JAMA benchmark criteria instrument is one of the leading tools used to evaluate medical information obtained from online sources.^16^^,^^17^ A score of 0 to 1 point represents insufficient information; 2 to 3 points, partially sufficient information; and 4 points, completely sufficient information.^16^^,^^17^

##### Global Quality Score

The GQS is a scoring system that can be used to assess a video in terms of its instructive aspects for viewers.^8^^,^^9^ A score of 1 point indicates that the video has the poorest quality and is not at all useful for viewers, whereas a score of 5 points indicates that the video has excellent quality and is very useful for viewers.^8^^,^^9^

### Statistical Analysis

Descriptive analyses were performed to examine the video sources; video content; type of video information; video characteristics; and video reliability, validity, and quality scores (i.e., DISCERN score, JAMA score, and GQS). Videos regarding anatomy, radiology, and patient experience were classified into a single category because of the restricted number of videos available for each category. Categorical variables are expressed as absolute frequencies with percentages. The normality of continuous variables was tested; normally distributed variables are presented as means and standard deviations, whereas non-normally distributed variables are presented as medians and ranges. Correlations between quantitative variables were estimated and tested using the Spearman correlation analysis. To assess whether outcomes—that is, video reliability, validity, and quality scores—differed by video information, 1-way analysis of variance was performed using the Bonferroni adjustment for multiple pair-wise comparisons. Radiology and patient experience were combined with anatomy because of low frequency.

## Results

**Fig 1:**
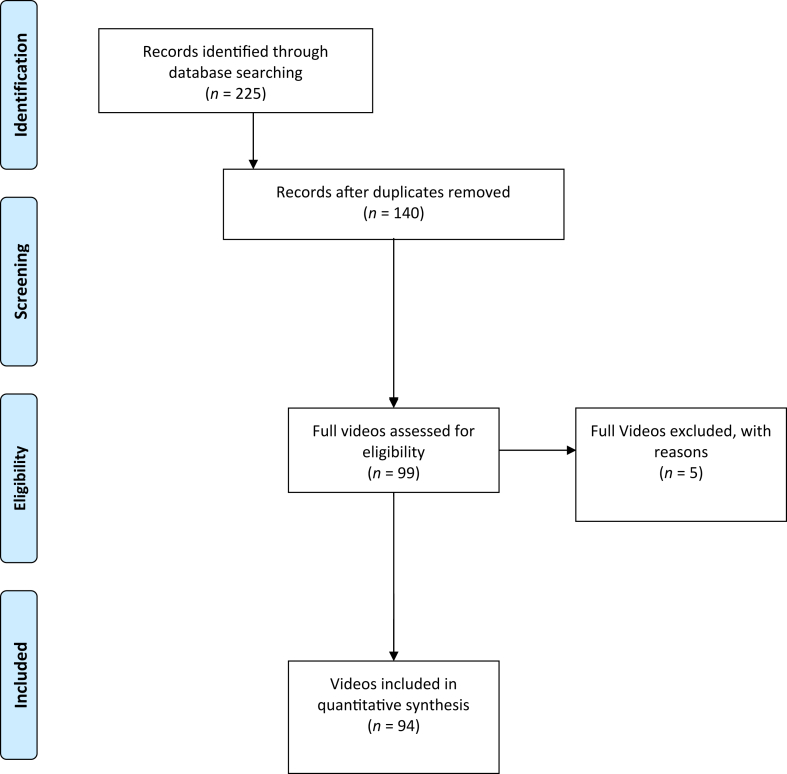
Flowchart of videos screening performed in study.

A total of 94 videos were included in the analysis (Fig 1). Of these videos, 88 (93.6%) were published by health professionals, whereas 4 (4.3%) were published by companies and 2 (2.1%) were published by private users. Most of the videos were about surgical technique (54%-57.4%), and almost all the videos (93%-98.9%) had an educational aim, with the exception of 1 video that reported a patient experience (1%-1.1%). The mean length of the videos was 648.4 ± 973.5 seconds, and the mean online period was 34.5 ± 27.0 months. The mean DISCERN score, JAMA score, and GQS were 32.9 ± 15.9, 1.5 ± 0.9, and 2.3 ± 1.0, respectively. Detailed results are reported in Table 1.

**Table 1:** Characteristics of Videos Included in Study

	Mean ± SD or n (%)	Median (Range)
Video source		
Health professional	88 (93.6)	
Company	4 (4.3)	
Private user	2 (2.1)	
Type of information		
Surgical technique	54 (57.4)	
Overview	21 (22.3)	
Anatomy	13 (13.8)	
Radiology	4 (4.3)	
Patient experience	2 (2.1)	
Video content		
Education	93 (98.9)	
Patient experience/testimony	1 (1.1)	
Video characteristics		
Months online	34.5 ± 27.0	24 (0.50-108)
Total No. of views	7,055.4 ± 33,325.3	267.50 (1-264,417)
Total No. of likes	112.4 ± 657.2	2 (0-4,879)
Total No. of comments	45.0 ± 27.4	0 (0-250)
Video length, s	648.4 ± 973.5	274.50 (7-5,732)
Video with ≥1 comment	33 (35.1)	
Video with ≥1 like	52 (55.3)	
Video score		
DISCERN	32.9 ± 15.9	25 (17-70)
JAMA	1.5 ± 0.9	1 (0-4)
GQS	2.3 ± 1.0	2 (1-5)

NOTE. A total of 94 videos were included in the analysis.

GQS, Global Quality Score; JAMA, *Journal of the American Medical Association*; SD, standard deviation.

### Correlations Between Quality of Videos and Information

All scores were directly positively correlated with video length (*P* < .001), and the JAMA score and DISCERN score were also positively correlated with the number of likes (*P* = .033 and *P* = .039, respectively). Detailed results are reported in Table 2.

**Table 2:** Correlations Between Scores (DISCERN, JAMA, and GQS) and Characteristics

	DISCERN		JAMA		GQS	
	ρ	*P* Value	ρ	*P* Value	ρ	*P* Value
Video length (in seconds)	0.82	<.001∗	0.78	<.001∗	0.67	<.001∗
Months online	–0.04	.715	–0.02	.871	–0.25	.016∗
Total No. of views	0.17	.102	0.17	.100	-0.06	.581
Total No. of likes	0.21	.039∗	0.22	.033∗	0.03	.748
Total No. of comments	0.14	.188	0.09	.372	0.00	.997

NOTE. A total of 94 videos were included in the analysis. ρ Values were estimated using Spearman rank correlation.

GQS, Global Quality Score; JAMA, *Journal of the American Medical Association*.

∗ Statistically significant.

### Correlations Between Variables

Positive correlations were found between the number of likes and the number of comments and video length, as well as between the period online and the number of comments, the number of likes, the number of views, and length (*P* ≤ .001). The number of views was positively correlated with the number of comments, number of likes (*P* < .001), and video duration (*P* = .024). Details are reported in Table 3.

**Table 3:** Correlations Between Variables

Variables	ρ	*P* Value
Total No. of likes and total No. of comments	0.68	<.001∗
Total No. of likes and video length (in seconds)	0.33	.001∗
Months online and total No. of comments	0.36	<.001∗
Months online and total No. of likes	0.36	<.001∗
Months online and total No. of views	0.51	<.001∗
Months online and video length (in seconds)	0.00	.978
Total No. of views and total No. of comments	0.59	<.001∗
Total No. of views and total No. of likes	0.82	<.001∗
Total No. of views and video length (in seconds)	0.23	.024∗

NOTE. A total of 94 videos were included in the analysis. ρ Values were estimated using Spearman rank correlation.

∗ Statistically significant.

### Score by Video Content

Videos that provided an overview of the ALL were the best in terms of all quality scores and were significantly higher quality than videos about surgical technique and anatomy, radiology, and patient experience for all the scores (*P* < .001). No difference was found between surgical technique and anatomy, radiology, and patient experience (DISCERN score, *P* > .99; JAMA score, *P* = .839; and GQS, *P* > .99). Details are reported in Table 4.

**Table 4:** Video Quality Based on Content

Type of Information	Mean ± SD	Pair-wise Comparisons (*P* Value∗)	
		Overview	Anatomy, Radiology, and Patient Experience
DISCERN score			
Surgical technique	25.3 ± 6.2	<.001†	>.99
Overview	57.0 ± 13.4		<.001†
Anatomy, radiology, and patient experience	27.8 ± 11.0		
JAMA score			
Surgical technique	1.1 ± 0.4	<.001†	.839
Overview	2.7 ± 0.9		<.001†
Anatomy, radiology, and patient experience	1.3 ± 0.9		
GQS score			
Surgical technique	1.9 ± 0.6	<.001†	>.99
Overview	3.5 ± 0.9		<.001†
Anatomy, radiology, and patient experience	2.0 ± 1.0		

NOTE. A total of 94 videos were included in the analysis.

GQS, Global Quality Score; JAMA, *Journal of the American Medical Association*; SD, standard deviation.

∗ Bonferroni adjustment was used for multiple comparisons.

† Statistically significant.

## Discussion

The main finding of this study is that YouTube videos about the ALL of the knee had poor reliability and validity and were found to be of low informational value. This was determined based on the DISCERN score, JAMA score, and GQS for the 94 videos analyzed. In line with our findings, most previous studies that have analyzed the quality and popularity of YouTube videos related to orthopaedic conditions in different subspecialties have reported low quality with respect to disease information, reliability, accuracy, and specific educational content.^8^, ^9^, ^10^, ^11^, ^12^

The emergence of COVID-19 accelerated the use of social media platforms such as YouTube, Twitter (San Francisco, CA), and Instagram (Facebook, Menlo Park, CA) to quickly share information about an unfamiliar and infectious disease and distribute this information directly to frontline journalists as new updates became available. This rapid and efficient dissemination of information illustrates the significant influence social media can have on the spread of medical literature and knowledge among health care professionals.^19^

The COVID-19 epidemic caused significant disruptions to medical education. It compelled medical schools and residency and fellowship training programs to adjust their methods of educating their students. With the help of virtual platforms such as Zoom (Zoom Video Communications, San Jose, CA) and Microsoft Teams (Redmond, WA), formal educational lectures, midday conferences, grand rounds, and medical conferences have transitioned to the internet to adjust to the current situation. Owing to the extensive cancellation of elective procedures, specialty training programs that focus on procedural skills encountered distinct difficulties in ensuring that their trainees would acquire sufficient proficiency in performing procedures. Fellowship programs implemented cutting-edge virtual training webinars to enhance participants’ theoretical knowledge in arthroscopy. They also used video sessions to review common technical aspects of surgery. Additionally, they revitalized the use of simulation-based training, which has demonstrated the transferability of skills learned in virtual reality simulation-based training to real-life scenarios. Whereas Zoom and Microsoft Teams have become popular virtual platforms for official medical education, informal medical education has long been available on many social media platforms such as YouTube. Furthermore, in the presence of social distancing measures, social media platforms facilitate the creation of a sense of community and camaraderie among health care personnel, which would not be possible otherwise.^19^

YouTube is the dominant video-sharing platform on the internet and serves as the primary free online resource for videos used by students and health care professionals globally. A survey conducted on a sample of 91 second-year medical students revealed that an overwhelming majority of 98% used YouTube as a Web-based source of information. On the creation of a YouTube channel for these medical students to enhance their comprehension of gross anatomy, 86% of the students used the channel, and of these individuals, 92% agreed or strongly agreed that the channel facilitated their learning of anatomy. YouTube is a highly successful tool for medical education that enhances trainee comprehension and integration of knowledge at both the molecular and clinical levels.^20^, ^21^, ^22^

Almost all publications dealing with orthopaedic conditions on YouTube have reported a high percentage of videos posted by health professionals, as was the case in our study (88 of 94 videos). YouTube has no editorial process for uploaded videos, and any user can upload any video of his or her choice, so it is plausible that this lack of restriction allows inaccurate video content to be published, albeit by health professionals. This is partly because of the lack of an editorial process and peer review on a platform such as YouTube. It also points to the need to involve communication professionals in medical communication to the general public, who can make the content uploaded online by doctors more accessible to the general public.

Rogers et al.^23^ aimed to determine the primary resources chosen by orthopaedic residents, as well as how they are used. Residents were asked to identify the top 3 online resources they used to stay current with and enhance fundamental orthopaedic knowledge. The responses were (1) Orthobullets (Santa Barbara, CA) (93%), (2) VuMedi (Oakland, CA) (44.5%), and (3) various websites (21.8%). The approach to enhancing general knowledge is to prioritize general websites, such as Orthobullets, and then move on to relevant textbooks, followed by relevant systematic reviews, meta-analyses, and general review articles.^23^

The high level of interest in ALL in recent years can be attributed to several factors. First, a media frenzy followed the “rediscovery” of the ALL. The news of the identification of this ligament, after the publication of the study by Claes et al.^24^ in 2013, was worldwide and reached media outlets of the size of *The New York Times*.^25^^,^^26^ This led to a high number of scientific studies analyzing the anatomy, biomechanics, and function of the ALL, resulting in an ever-increasing number of publications on this ligament. The clinical results of the addition of reconstruction of this ligament concomitant with ACLR did not take long to appear.^27^ Benefits such as better clinical outcomes, decreased residual laxity, a lower rate of graft failure, and a reduced rate of reoperations compared with isolated ACLR were reported by study groups all over the world.^28^, ^29^, ^30^, ^31^, ^32^, ^33^, ^34^ This has opened the door to the rediscovery of LET in combination with ACLR to reduce the risk of graft rupture and improve the results.^35^ In particular, several comparative clinical studies, including a randomized controlled trial,^36^ have shown lower primary anterior cruciate ligament graft rupture rates with combined reconstruction. This is particularly true in high-risk populations such as young active patients participating in contact or pivoting sports,^37^ patients with chronic anterior cruciate ligament injuries,^38^ pediatric patients,^39^^,^^40^ patients with hyperlaxity,^41^ and professional athletes.^42^ The significant reductions in graft rupture rates observed in numerous studies at short- to medium-term follow-up are maintained at long-term follow-up.^43^ As a result, lateral extra-articular procedures, whether ALL reconstruction or LET, are no longer niche procedures and their use is of interest to surgeons and patients around the world.

Similar to the results of our study, Çelik et al.^44^ and Yüce et al.^45^ reported a positive correlation between the quality and duration of videos. This can be interpreted to mean that longer videos allow content creators to explain the condition in more detail. As a result, longer videos are more reliable and valid and of higher quality, so they are more appreciated by the general audience.

Recently, D’Ambrosi et al.^11^ assessed the educational quality of YouTube videos regarding ramp lesions of the meniscus, finding that the educational content of YouTube videos about medial meniscal ramp lesions showed low quality and validity. A similar study analyzed the validity and informational value of teaching material regarding ACLR using quadriceps tendon (QT) autograft provided on the YouTube video platform.^12^ The study revealed that YouTube is a fast and open-access source of mass information. The overall quality of the videos on ACLR performed using QT autograft was unsatisfactory, showing low educational quality and reliability and confirming that YouTube cannot be recommended as a reliable source of information on ACLR with QT.^12^ Patients and physicians frequently use YouTube as a source of information, and the results of our study emphasize the need for higher-quality orthopaedic educational content for viewers and patients on this video platform.

### Limitations

There are several limitations to this study. First, keyword searches on YouTube may not have yielded all videos on the platform that address the topic. However, we were careful to use a variety of keywords and believe that we have likely included all possible terms describing this entity. Variables such as geographic location or user characteristics may also affect the results of YouTube’s search algorithm. Surgical videos may be omitted because the platform requires a registered user of legal age to view them. Non–English-language videos were excluded from the analysis, which further reduces the generalizability of our findings. Finally, validated instruments to assess the quality of health information in videos do not exist. This study used tools designed to assess reliability, validity, and quality, such as the DISCERN tool, JAMA criteria, and GQS, which have not been validated. However, these tools are widely used in studies that are evaluating these measures for online resources.

## Conclusions

The educational content of YouTube videos about the ALL of the knee showed low quality and validity based on the DISCERN score, JAMA score, and GQS.

## Disclosures

The authors declare the following financial interests/personal relationships which may be considered as potential competing interests: B.S-C. declares to be a consultant for Arthrex. All other authors (R.D., A.C., A.A., T.D.V., J-S.A., B.F.) declare that they have no known competing financial interests or personal relationships that could have appeared to influence the work reported in this paper.
